# Gypsogenin Battling for a Front Position in the Pentacyclic Triterpenes *Game of Thrones* on Anti-Cancer Therapy: A Critical Review—Dedicated to the Memory of Professor Hanaa M. Rady

**DOI:** 10.3390/molecules28155677

**Published:** 2023-07-27

**Authors:** Mohamed O. Radwan, Howaida I. Abd-Alla, Azhaar T. Alsaggaf, Hatem El-Mezayen, Mohammed A. S. Abourehab, Mohamed E. El-Beeh, Hiroshi Tateishi, Masami Otsuka, Mikako Fujita

**Affiliations:** 1 Medicinal and Biological Chemistry Science Farm Joint Research Laboratory, Faculty of Life Sciences, Kumamoto University, Kumamoto 862-0973, Japan; 2 Chemistry of Natural Compounds Department, National Research Centre, Giza 12622, Egypt; 3 Department of Chemistry, Taibah University, Madinah 42353, Saudi Arabia; atsagaaf@taibahu.edu.sa; 4 Biochemistry Department, Helwan University, Cairo 11795, Egypt; hatem_mezayen@science.helwan.edu.eg; 5 Department of Pharmaceutics and Industrial Pharmacy, Faculty of Pharmacy, Minia University, Minia 61519, Egypt; 6 Department of Pharmaceutics, Faculty of Pharmacy, Umm Al-Qura University, Makkah 21955, Saudi Arabia; 7 Biology Department, Al-Jumum University College, Umm Al-Qura University, Makkah 21955, Saudi Arabia; 8 Department of Drug Discovery, Science Farm Ltd., Kumamoto 862-0976, Japan

**Keywords:** pentacyclic triterpenes, gypsogenin, anti-cancer

## Abstract

In the last decade, gypsogenin has attracted widespread attention from medicinal chemists by virtue of its prominent anti-cancer potential. Despite its late identification, gypsogenin has proved itself as a new anti-proliferative player battling for a frontline position among other classic pentacyclic triterpenes such as oleanolic acid, glycyrrhetinic acid, ursolic acid, betulinic acid, and celastrol. Herein, we present the most important reactions of gypsogenin via modification of its four functional groups. Furthermore, we demonstrate insights into the anti-cancer activity of gypsogenin and its semisynthetic derivatives and go further by introducing our perspective to judiciously guide the prospective rational design. The present article opens a new venue for a better exploitation of gypsogenin chemical entity as a lead compound in cancer chemotherapy. To the best of our knowledge, this is the first review article exploring the anti-cancer activity of gypsogenin derivatives.

## 1. Introduction

Cancer is the second major global cause of mortality preceded with cardiovascular diseases [1,2,3,4]. Having said that, cancer cases are soaring at an alarming worldwide rate. Surprisingly, in some countries, cancer has exceeded cardiovascular disorders as a leading mortality cause [5]. This horrifying fact has been discussed in a cohort study that pointed out a transition in the main causes of deaths among youth in some countries [6]. Medicinal chemists are continuously urged to innovate new chemical entities to surmount resistance, reduce side effects, and enhance the efficacy of commercial drugs in the hard-fought battle against cancer [7,8,9,10,11,12]. Many natural products have provided skeletons and structural references for the invention of modern drugs [13,14,15,16]. Found in higher plants, pentacyclic triterpenes (PTs) are bio-nutrient phytochemicals endowed with a diverse range of bioactivities such as hepatoprotective [17,18,19], anti-inflammatory [20,21,22], anti-hypertensive [19,23,24,25], anti-atherosclerotic [23,24], anti-viral [26,27,28], anti-fibrosis [29,30,31], and anti-ulcer effects [20,32,33]. In particular, PTs have ubiquitous applications in terms of anti-cancer drug discovery [34,35,36,37,38,39,40,41,42].

**Figure 1:**
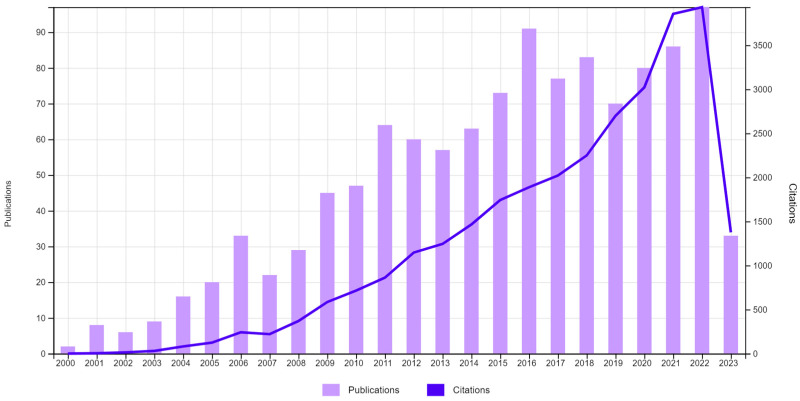
Number of citations and scientific publications containing research linking triterpenes with anti-cancer activity over the period 2000–2023. Data were obtained from the Web of Science database by searching for the keywords triterpene cancer.

The literature is loaded with plenty of success stories linking PTs derivatives with a prominent role in the prevention of cancer initiation, promotion, angiogenesis, and progression through disrupting different intermittent mechanisms and pathways. The number of scientific publications and citations linking PTs and cancer has been soaring over the past twenty years, according to the Web of Science database (Figure 1). PTs are generally non-cytotoxic, albeit minor derivatizations can lead to dramatic changes in activity.

PTs comprise four main chemical skeletons, namely oleanane, ursane, lupane, and friedelane. Oleanolic acid, from the oleanane type, is one of the most extensively studied PTs in terms of medicinal chemistry. Oleanolic acid suppresses proliferation of hepatocellular carcinoma [42,43], human bladder cancer [44], breast cancer [45,46], lung carcer [47], and colon cancer [48,49]. To possess such diverse activities, oleanolic acid modulates multiple cell-signaling pathways [50]. Two oleanolic acid derivatives, CDDO and CDDO-Me, have already entered clinical trials for the treatment of solid tumors and lymphoma, allowing oleanolic acid to top the throne of pentacyclic triterpenes in terms of chemotherapy [51,52]. Glycyrrhetinic acid is another representative of oleanane-type triterpenoids with ubiquitous anti-cancer activities [53,54,55,56,57,58]. Ursolic acid [48,59,60,61,62], betulinic acid [63,64,65,66,67,68], and celastrol [69,70,71,72], representing ursane, lupane, and friedelane type triterpenoids, respectively, were reported to possess multifaceted anti-cancer properties.

**Figure 2:**
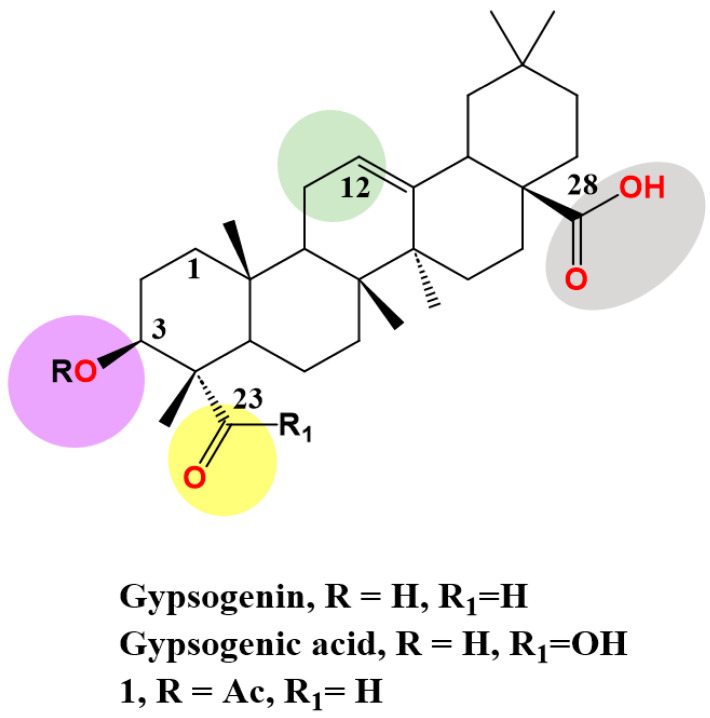
Structure of gypsogenin, gypsogenic acid and 3-acetyl gypsogenin (**1**) highlighting the four functional groups.

Gypsogenin (3-hydroxy-23-oxoolean-12-en-28-oic acid), a less-explored PT, extracted from *Gypsophila oldhamiana* in a saponin form linked with sugar moieties. It is generated as pure sapogenin via acid hydrolysis [73]. It has an oleanane-type skeleton and possesses four active sites, C-3 hydroxyl, ring C double bond, C-23 aldehyde group and C-28 carboxylic acid, which are amenable to a wide range of chemical transformations (Figure 2). The hydroxyl, alkene, and carboxyl groups exist in most PTs. Nevertheless, the aldehyde group is unique, as other classic triterpenes lack such a group, which represents a structural alert for most medicinal chemists due to its high reactivity [74].

Previously, aldehydes used to have an unfavorable reputation due to their toxicity and metabolic instability. Nonetheless, in modern chemical biology, they have been applied as covalent probes to target lysine residues in proteins by forming a covalent imine adduct. In this regard, roblitinib development as exquisitely selective inhibition of FGFR4 signaling was based on the presence of an aldehyde group. The latter is responsible for creating a reversible-covalent bond with the target while avoiding the safety concerns of irreversible covalent inhibitors [75]. Taken together, the aldehyde group will play an important role in drug discovery in the 21st century to find ligands for traditionally undruggable targets [74,76]. This may give gypsogenin and advantage over other PTs.

Recently, gypsogenin proved itself as an outstanding entity that can enter the competition between PTs for a frontline position as a lead anti-cancer agent. Most previous reports linked gypsogenin to anti-cancer effects. It is unlikely that other bioactivity will be found for gypsogenin and its derivatives; one example is the observed strong inhibition of acetylcholinesterase, which provides a basis for potential Alzheimer’s therapy involving natural products [77]. Stunningly, the first carboxamide series of gypsogenin came out in 2018, which points out the shortage of structure–activity relationship (SAR) studies on this precious PT [73]. Moreover, no gypsogenin derivatives with modified ring C were synthesized before 2023.

Several PTs exhibit limited water solubility and low bioavailability, which can be addressed by derivatization [78]. Derivatization not only optimizes triterpenes’ pharmacokinetics, but also their pharmacodynamics. Herein, we summarized the chemical modifications of gypsogenin four functional groups and focused on the anti-cancer effect of gypsogenin and its semi-derivatives. We generated SAR for gypsogenin and its derivatives against leukemia, breast cancer, and lung cancer. We present our recommendations for prospective work and the missing information that should be addressed. Our study represents a cornerstone reference for any future research linking gypsogenin and cancer. We believe that future extensive SAR studies of gypsogenin will advance it to a frontline position in the pentacyclic triterpenes *Game of Thrones* on anti-cancer therapy.

## 2. Methodology

This review article is the first to discuss gypsogenin and its derivative from a medicinal chemistry perspective. We used the keywords gypsogenin derivative and anti-cancer for our search in PubMed and Web of Science. This disclosed approximately 60 articles and patents, of which 27 were considered for this review. As this study focuses on medicinal chemistry aspects, we excluded the anti-cancer activity of the naturally found gypsogenin saponins and considered the semi-synthetic derivatives of gypsogenin for this review.

## 3. Gypsogenin Extraction and Chemical Transformation

The difficulty of isolation of gypsogenin from plants and the high price of commercially available gypsogenin limited extensive SAR studies. One extraction example showed that starting with 20 kg of air-dried roots of *Gypsophila oldhamiana* yields as little as 1.3 g of pure gypsogenin. The procedures were initiated via water extraction of the water-soluble saponins before drying under a vacuum. The mixture was subjected to acid hydrolysis using 10% HCl for 72 h before neutralization with NaOH and extraction with ethyl acetate. After evaporation, the mixture was applied to column chromatography using 10:1 hexane–ethyl acetate eluent to give rise to gypsogenin as a white solid [73,79,80]. Gypsogenin can also be found in other species of *Gypsophila*, such as *bermejoi*, *simonii* [81], *paniculate*, and *arrostii* [82]. Additionally, it is available in plants belonging to the Caryophyllaceae family, such as *Agrostemma githago* (*Lychnis githago*) [83,84], *Melandrium firmum* [85], and different *Stellaria* species [86,87]. Furthermore, plants that belong to the Amaranthaceae family, e.g., *Beta vulgaris* L [88] and *Chenopodium quino* [89], contain gypsogenin. Greatrex et al. synthesized gypsogenin from the alcoholic PT analogue, hederagenin, via oxidation [90].

As we mentioned above, gypsogenin has four functional groups that can be feasibly modified to enhance its pharmacodynamic and pharmacokinetic profile. The 3-OH group was acetylated using the conventional method used for other PTs—reflux with acetic anhydride in dry pyridine—as described by Emirdag et al. [91]. The addition of dimethyl amino pyridine (DMAP) as a catalyst was used elsewhere to improve yield [77,92]. The 3-OH group was recently oxidized, forming the 3-keto analogue. This was achieved by mixing gypsogenin with Dess–Martin periodinane in dichloromethane at 0 °C for 15 min [92]. The authors also reported 3-OH etherification using different alkyl bromides in the presence of potassium iodide and potassium carbonate in dimethyl formamide (DMF) at 60 °C [92]. Dehydration of gypsogenin by thionyl chloride in (DMF) eliminates the 3-OH group and produces its unsaturated 2,3 dehydro- analogue [92].

Gypsogenic acid (Figure 2), the dicarboxylic acid analogue of gypsogenin, can be isolated from *Gypsophila oldhamiana* roots, especially if a portion of gypsogenin is transformed into gypsogenic acid during the hydrolysis step. In addition, its 3-acetyl analogue was synthesized through oxidation of 3-acetyl gypsogenin (**1**) by sodium hypochlorite and hydrogen peroxide in the presence of sodium dihydrogen phosphate at room temperature [93]. A similar oxidation process could be achieved via vigorous stirring with potassium permanganate in ethanol water mixture at room temperature [93].

**Figure 3:**
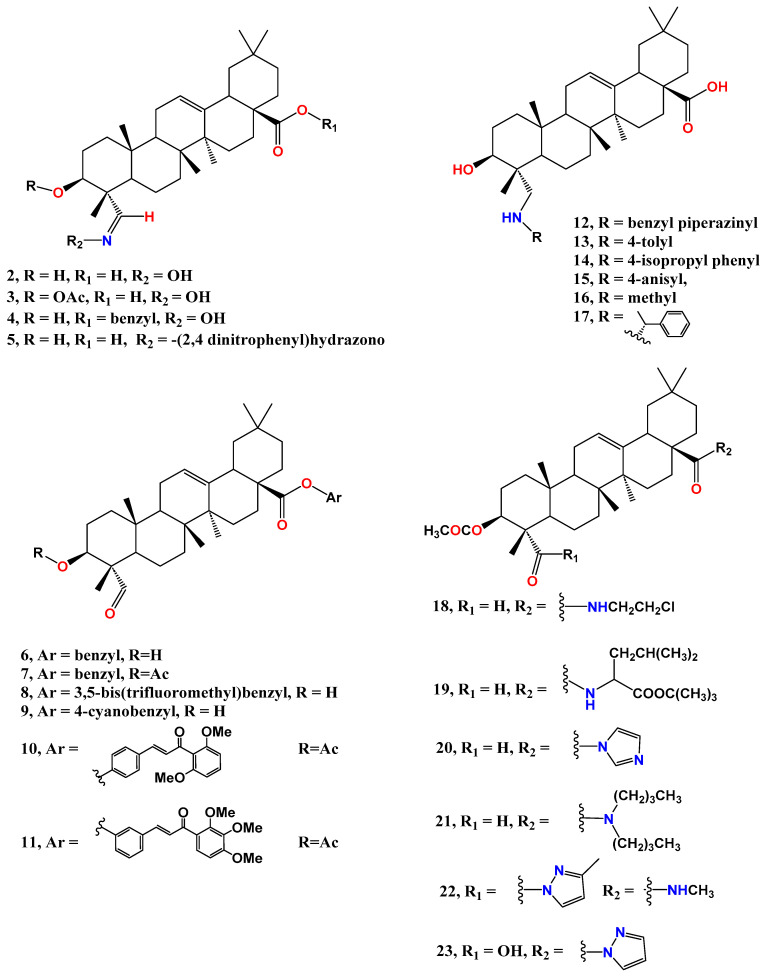
Structure of gypsogenin and gypsogenic acid bioactive derivatives through reaction with 3-OH, C-23-CHO or -COOH, and C28-COOH.

The 4-aldehyde group of gypsogenin is versatile and has been reacted in different ways. Its oximation by using hydroxylamine hydrochloride in pyridine at 105 °C afforded compound **2** in a good yield (Figure 3) [73,91]. It was also reacted with thiosemicarbazide in a 1:1 MeOH: water mixture under reflux forming a thiosemicarbazone analogue [91]. Another amination of gypsogenin’s 4-aldehyde was performed in acetic acid using phenyl hydrazine or 2,4-dinitrophenylhydrazine solvent at room temperature; the latter resulted in the formation of Schiff base **5** [73].

We have performed reductive amination of gypsogenin’s 4-aldehyde group using different amines and sodium triactoxyborohydride for in situ reduction of the formed Schiff base in dichloroethane solvent at room temperature (compounds **12**, **13**, **14**, **15**, and **17**) [94,95]. The yield of this reaction was generally poor due to the low solubility of gypsogenin in dichloroethane. That is why another group performed this reaction in methanol while using sodium borohydride as a reducing agent to obtain compound **16** [92].

The third functional group of gypsogenin is 28-COOH, which is widely found in PTs. A feasible esterification process involves activation by potassium carbonate in DMF at room temperature, followed by addition of appropriate alkyl bromide. This was applied for synthesis of **6** [95], **8**, and **9 [96]** in good yields. Hybrids of gypsogenin and chalcones achieved via ester bond were disclosed in a recent patent [79]. Esterification of 3-acetyl gypsogenin with different substituted chalcones was achieved using *N*, *N*′-Dicyclohexylcarbodiimide (DCC) in the presence of 4-dimethylaminopyridine compounds **10** and **11** (Figure 3).

Different amides of 3-acetyl gypsogenin were produced via activation of the carboxyl group with oxalyl chloride, followed by addition of the appropriate amine in the presence of triethyl amine as a catalyst in dichloromethane [73,92]. This general method was applied for the synthesis of the amides shown in Figure 3, such as compounds **18**, **19** [92], and **20** [93] in good yields. Bisamidation was performed for 3-acetyl gypsogenic acid, adopting the same procedures to obtain derivatives such as **22** through reaction with two different amines for each carboxyl group [93]. Some reported bisamides were synthesized by reacting dichloride of gypsogenic acid with the two molar equivalents of the same appropriate amine [77,93].

**Figure 4:**
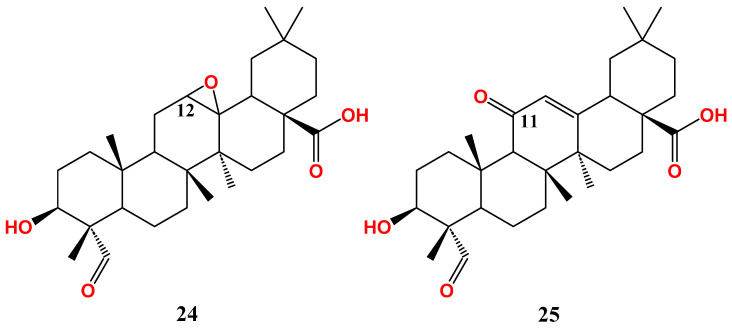
Gypsogenin derivatives with modified ring C.

Facile oxidation approaches of ring C were recently conducted using different conditions resulting in different products. Stirring of gypsogenin with hydrogen peroxide and formic acid in dichloromethane at room temperature afforded the epoxide congener (**24**). On the other hand, oxidation of gypsogenin using selenium dioxide in acetic acid under reflux gave rise to the 11-keto derivative (**25**) [92] (Figure 4). The produced enone system of ring C imitates that naturally found in glycyrrhetinic acid. The molecular formula and molecular weight of the compounds in Figure 2, Figure 3 and Figure 4 were summarized in Table S1 (Supporting data).

## 4. Anti-Cancer Effect of Gypsogenin, Gypsogenic Acid, and Their Semisynthetic Derivatives

### 4.1. Anti-Leukemic Activity

Gypsogenic acid did not show observable activity against chronic myeloid leukemia (K562) and acute myeloid leukemia (HL-60), where its IC_50_ exceeded 100 µM for both cells [97]. Another study was in accordance with this, recording the activity of gypsogenic acid IC_50_ against K562 as 227.6 µM; however, HL-60 was more sensitive (IC_50_ 61.1 µM) [98]. The latter value shows a discrepancy with the previous report by Lee’s group [97]. Gypsogenic acid demonstrated low activity against other lymphoid leukemias SKW-3 and BV-173 (IC_50_ 79.1 and 41.4 µM, respectively) [98].

Later on, we found that gypsogenin highly outperforms gypsogenic acid with IC_50_ 12.7 µM against K562, highlighting the crucial role of the 4-aldehyde group [96]. Simultaneously, Emirdag et al. revealed that gypsogenin has anti-proliferative effect on HL-60 (IC_50_ 10.4 µM) by inducing apoptosis [82,91]. 3-acetyl gypsogenin, **1**, has almost the same effect of gypsogenin on HL-60 (IC_50_ 10.77 µM). Gypsogenin activity is increased by oximation of its aldehyde group (compound **2** IC_50_ 3.9 µM). Mutually, the 3-acetylated oxime analogue **3** surpassed the activity of **1** (IC_50_ 5.9 µM) [91]. Gypsogenin benzyl ester **6** has IC_50_ 8.1 µM; however, its acetylation product **7** has IC_50_ 6.7 µM [91].

By virtue of its notable apoptotic effect, **6** was further benchmarked for its effect on K562 cell line, where it showed moderate activity (IC_50_ 9.3 µM) [99]. However, this study represented a turning point for a better understanding of gypsogenin’s molecular target. Compound **6** inhibited ABL1 tyrosine kinase with IC_50_ 8.71 µM. This is assumed to be the main target for its cytotoxic effect on K562. It is needless to say that the presence of other off targets cannot be excluded. Concomitantly, **6** inhibited other kinases such as C-terminal Src kinase (CSK) and Lyn kinase isoform B; LYN B (IC_50_ 1.5 µM and 2.9 µM, respectively) [99]. It is clear that oximation of **6** is detrimental for its activity on both K562 and HL-60, as the respective IC_50_ value of **4** is 21.3 µM and 10.6 µM [99].

Ciftci et al. moved forward with a structure–activity relationship study of **6** and succeeded in enhancing its activity [96]. As mentioned above, the free aldehyde group is crucial for activity against leukemia. Therefore, Ciftci et al. came up with substituted congeners of 6, keeping a free 4-aldehyde group [96]. Compounds **8** and **9** have IC_50_ 4.7 and 3.1 µM, respectively, against K562 cells. Additionally, IC_50_ of **8** and **9** for ABL1 tyrosine kinase was 7.1 µM and 6.1 µM, respectively. Both compounds have induced an explicit apoptosis effect, especially **8**, whose apoptosis induction was clearer than imatinib, a gold standard ABL1 kinase inhibitor for CML therapy. Concomitantly, 8 suppressed the downstream signaling of extracellular signal-regulated kinase (ERK) phosphorylation [96]. In a similar vein, both compounds exhibited moderate activity on MT-2 and Jurkat cells. Interestingly, the IC_50_ of 9 for MT-2 and Jurkat was 7.2 µM and 4.8 µM, respectively. The authors evaluated both compounds to determine their cytotoxic effect on peripheral blood mononuclear cells (PBMC) and calculated the selectivity index as the ratio of IC_50_ between PBMC and K562 cells. The higher selectivity index value of compound **8**, 11.0, than compound **9**, 8.0, reflects the favorable safety profile of compound **8**.

**Figure 5:**
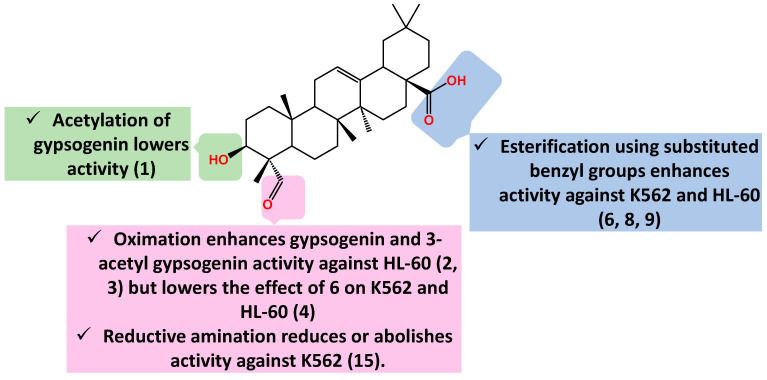
Summary of gypsogenin derivatives SAR pertaining to cytotoxicity against K562 and HL-60 cells.

A recent report by Ulusoy et al. showed that reductive amination of the 4-aldehyde group with different aromatic and alicyclic amines leads to either reduction or complete abrogation of anti-K562 activity [95]. The hit compound in this study, **13**, had IC_50_ 11.3 µM which is even less active than the parent compound, gypsogenin [95]. Furthermore, **13** inhibited ABL1 kinase in a moderate fashion (IC_50_ value of 13.0 µM). This is further evidence of the crucial role of the 4-aldehyde group for anti-K562 activity (Figure 5). In addition, **13** had less effect on MT-2 and Jurkat than **8** and **9**. Compound **13** had moderate effect on a panel of kinases at 30 µM of drug concentration, especially for BRK, BTK, LYN B, and SRC. Compound **14** with the more hydrophobic 4-isopropyl substitution exhibited less activity (IC_50_ 23.8 µM), whereas the presence of a bulky *N*-piperazinyl benzyl moiety abolished activity as shown for **12** (IC_50_ > 100 µM). The activity was also abolished in the presence of an electron-donating substitution, as was the case for **15** (IC_50_ > 100 µM).

So far, there has been no report linking gypsogenin or gypsogenic acid carboxamides and leukemia. This is the same case for modified ring C derivatives and gypsogenin–chalcone hybrids. In a word, gypsogenin benzyl esters have been the most active derivatives against K562 and HL-60 leukemias until now. The SAR pertaining to activity against K562 and HL-60 is afforded in Figure 5.

### 4.2. Anti-Breast Cancer Activity

Gypsogenin has moderate cytotoxic activity for MCF-7 (IC_50_ 9.0 µM); however, its benzyl ester derivative 6 has IC_50_ 5.1 µM [91]. Surprisingly, substituted benzyl esters such as 8 and 9 showed less activity than gypsogenin with respective IC_50_ 51.58 µM and 15.3 µM. Notably, the 3-acetyl analogues 1 and 7 possess less activity (IC_50_ 20.5 µM and 65.1 µM, respectively). However, oximation of gypsogenin and **6** slightly improves their cytotoxic effect, as shown for 2 and 4. The exact mechanism of action is yet to be elucidated [91]. Notably, compound **1** has low IC_50_ value of 5.4 µM against triple-negative breast cancer cell (TNBC) line (MDA-MB-231). In this regard, two gypsogenin–chalcone hybrids demonstrated moderate effect, too, namely, **10** and **11** with respective IC_50_ 11.0 µM and 7.9 µM [79]. This can be a clue for targeting TNBC, which is an aggressive form of breast cancer that does not respond to hormonal therapy [100].

Wu et al. found that gypsogenic acid has a weak antiproliferative effect on MCF-7 (IC_50_ 26.8 µM), which also highlights the role of the 4-aldehyde group. The authors highly enhanced gypsogenin and gypsogenic acid activity through mono-and bisamidation [93]. Gypsogenin carboxamide with imidazole, compound **20**, has IC_50_ 3.7 µM, which is similar to the gypsogenic acid mono-amide of only C28 with pyrazole, compound **23,** whose IC_50_ is 3.8 µM. Gypsogenic acid bisamide of both C23 and C28, compound, **22** demonstrated pronounced activity (IC_50_ 4.1 µM). The favorable safety profile of those carboxamides is shown by measuring their activity on human umbilical vein endothelial cells (HUVEC cells). It was determined that **22** possesses the highest selectivity index (24.0) among the mentioned active compounds.

Further evidence of the efficiency of gypsogenin amides was disclosed this year by Sun et al. [92]. Two amides, **18** and **19**, possess IC_50_ 5.7 µM and 13.8 µM, respectively, towards MCF-7. They also synthesized compound **16** via reductive amination reaction using methylamine; its IC_50_ is 11.3 µM, which is greater than that of gypsogenin (IC_50_ 9.0 µM). The selectivity index of **16**, **18**, and **19** exceeds 30 when related to their effect on HUVEC.

**Figure 6:**
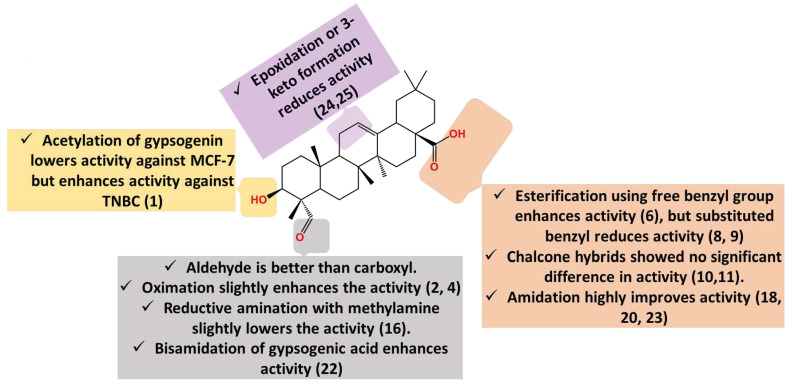
Summary of gypsogenin derivatives SAR pertaining to cytotoxicity against breast cancer cells.

Ring C-oxidized gypsogenin derivatives have recently been developed (Figure 4) [92]. The epoxide derivative (**24**) has IC_50_ 26.6 µM on MCF-7. In parallel with this, the 11-keto derivative (**25**) has similar activity (IC_50_ 25.3 µM), implying that oxidation of ring C reduces MCF-7 sensitivity. Conclusively, gypsogenin carboxamides are the best cytotoxic entities against MCF-7 compared to other derivatives (Figure 6).

### 4.3. Anti-Lung Cancer Activity

Gypsogenin can inhibit the growth and metastasis of Lewis lung cancer through inhibition of tumor angiogenesis and induction of apoptosis [101]. Different molecular targets were implicated in this mechanism. Gypsogenin downregulated mutant P53 and vascular endothelial growth factor (VEGF). It reduces the expression of Bcl-2 protein and raises Bax expression, promoting tumor apoptosis. The anti-proliferative effect of gypsogenin, (**1**), and 3-acetyl gypsogenic acid against A549 lung cancer cells is moderate (IC_50_ 19.6, 30.8, and 23.7 µM, respectively) [73,93]. Oximation of gypsogenin and **1** maintains the activity without significant change [73]. 2,4-dinitrophenyl)hydrazono derivative of gypsogenin (**5**) demonstrated a strong cytotoxic effect on A549 cells (IC_50_ 3.1 µM) [73,80]. In accordance, the amino product (**16**) exhibited stronger cytotoxic effect (IC_50_ 1.5 µM) [92].

The two carboxamides **20** and **23** showed a bit higher activity than compound **5** (IC_50_ 2.5 and 2.8 µM, respectively) [93]. Both compounds destroyed the cell membrane and increased its permeability, leading to the outflow of intracellular nucleic acid, but they weakly induced apoptosis and arrested A549 cell cycle of [93]. Another anti-lung cancer hit is the gypsogenic acid bisamidation product of (**22**), whose IC_50_ value is 2.0 µM. However, it is noteworthy that mono-amidation products **20** and **23** surpass its activity but with a lower selectivity index for HUVEC.

Concomitantly, compounds **18** and **19** showed a sub-micromolar effect on A549 (IC_50_ 0.5 µM and 0.9 µM, respectively) and induced both apoptosis through damaging the cell membrane and arresting the cell cycle. Combining in silico and in vitro tools defined VEGF1 as a gypsogenin target [92]. Remarkably, compound **18** showed a higher binding affinity to VEGF1 than the parent compound, which is in accordance with the cytotoxicity results. Gypsogenin esters showed disappointing results, such as those found for **8**, whose IC_50_ exceeds 100 µM and **9** which is less active than the parent compound (IC_50_ 24.5 µM). On the contrary, esterification with chalcone moieties elevated A549 sensitivity; the IC_50_ of **10** and **11** is 4.9 µM, and 1.3 µM, respectively [79]. This result denotes the role of chalcone moiety in conferring gypsogenin with high activity.

**Figure 7:**
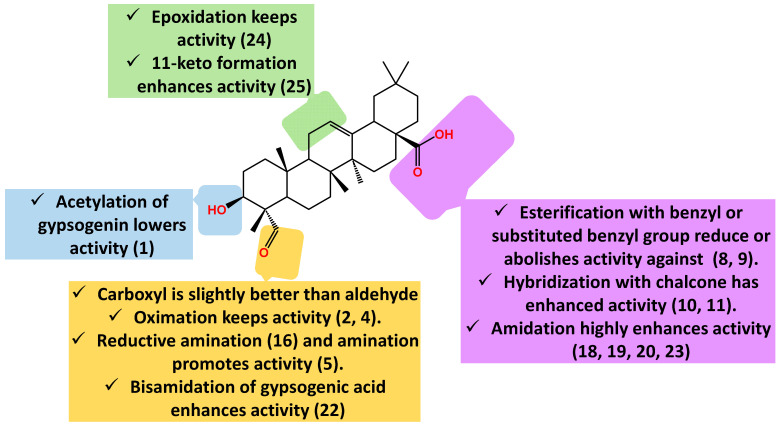
Summary of gypsogenin derivatives SAR pertaining to cytotoxicity against lung cancer cells.

The epoxide analogue (**24**) has almost the same activity as the parent compound (IC_50_.18.7 µM), whereas the 11-keto derivative (**25**) has slightly better activity (IC_50_.13.5 µM) [92]. In conclusion, gypsogenin carboxamides and chalcone hybrids are the most promising anti-proliferative entities against A549 (Figure 7).

### 4.4. Other Anti-Cancer Activities

A batch of gypsogenin derivatives demonstrated other notable anti-cancer effects. In this regard, we will focus mainly on compounds with at least single-digit micromolar IC_50_ values. Gypsogenin and its 3-acetyl form (**1**) possess remarkable cytotoxic activity against HeLa (cervical cancer) [79]. Compound **2** has notable anti-proliferative activity against SaoS-2 cells (osteosarcoma) and HeLa cells. Its 3-acetylated derivative (**3**) also has a similar effect on SaoS-2 but not on HeLa. It is noteworthy that gypsogenin has IC_50_ 7.8 against SaoS-2 which is better than **1**, **2**, and **3**. On the other hand, **3** is distinguished by its prominent activity against HT-29 cells (colorectal adenocarcinoma) [91] (Table 1).

Another study showed that gypsogenin can suppress gastric cancer cells NCI-N87 proliferation by targeting VEGF and MM-9 and promoting the expression of caspase-3 and Bax proteins [102]. Compounds **5** and **21** were reported mainly for targeting colon cancer cells (LOVO) through strong induction of apoptosis and dose-dependent S-phase arrest in cells. Both compounds exhibited moderate effect on SKOV3 (ovarian cancer) and HepG2 cells (Hepatocellular carcinoma) [73]. The amino compound **16** also exhibited notable activity against LOVO. Compounds **2** and **3** showed no or moderate activity towards LOVO [92]. The most active compound against LOVO cells is compound **8** with submicromolar cytotoxicity, implying that gypsogenin carboxamides usually outperform other derivatives [92] (Table 1).

Three amides were reported by Wu et al., **20**, **22**, and **23** with outstanding activities against HepG2, TE-1 (esophageal cancer), and MC3-8 (colon cancer) cells [93]. Gypsogenin–chalcone hybrids **10** and **11** showed outstanding activity against HeLa and pancreatic cancer cells (PANC-1). Gypsogenin 28-COOH ester **9** showed better activity in HeLa cells than **8 [96]**. Ciftci et al. revealed new derivatives that suppress glioma proliferation through EGFR inhibition. The amino derivative compound **17** has the strongest effect against EGFR and glioma cells U251, T98G, and U87 (Table 1). Consequently, the titled compound clearly induced apoptosis of U251 cells in a comparable fashion to cisplatin. This study revealed that gypsogenin benzyl esters were less effective than **17** on glioma cells [94] (Table 1). Furthermore, at 30 µM concentration, compound **17** showed moderate inhibition for a panel of other kinases, including ABL1 tyrosine kinase.

## 5. Conclusions and Future Directions

Befitting its anti-cancer promise, we presented a critical review of gypsogenin and its derivatives. Gypsogenin possesses a versatile and unique aldehyde group that can be utilized to create covalent interactions with undruggable targets. We dissected how gypsogenin was employed for semi-synthesis by reacting its four functional groups, then we demonstrated the bioactivity of the most important derivatives in the literature. So far, gypsogenin carboxamides have demonstrated high cytotoxic activity against breast and lung cancer. The bisamides of gypsogenic acid possess prominent activity as well; however, their anti-leukemic activity is yet to be explored. Gypsogenin benzyl esters showed pronounced activity against CML. Ring C-modified gypsogenin derivatives are weak antiproliferative agents against lung and breast cancer, but they have not been tested for their anti-leukemic effect. Gypsogenin and its derivatives were reported to target kinases such as ABL1 and VEGF. The selectivity index of some active compounds is high, reflecting their potential high safety. Further medicinal chemistry studies on gypsogenin are urgently needed to afford more active hits and elucidate their other plausible molecular targets.

## Figures and Tables

**Table 1:** Gypsogenin derivatives with different cytotoxic activities.

Compound		Cell Line and IC_50_ Value (µM)	
	HT-29 [92]	Saos-2 [82]	HeLa [91]
Gypsogenin	10.4	7.8	22.4
1	11.1	8.2	35.0
2	10.8	7.9	8.7
3	6.7	8.9	>100
		LOVO [93]	
16		5.8	
18		7.2	
19		0.8	
24		>30	
25		17.8	
	LOVO [74]	HePG2 [73]	SKOV3 [73]
5	2.9	10.0	9.7
21	3.5	12.5	13.1
	HepG2 [94]	TE-1 [93]	MC3-8 [93]
20	4.0	4.7	2.9
22	3.6	5.4	4.8
23	2.2	4.2	2.6
		HeLa [80,97]	PANC-1 [80]
Gypsogenin		9.4	13.5
1		3.3	5.0
8		35.2	-
9		5.6	-
10		9.5	8.7
11		10.2	7.9
U251		T98G	U87
17 [95]	5.8	8.1	17.0

## Data Availability

Not applicable.

## Supplementary Materials

The following supporting information can be downloaded at: https://www.mdpi.com/article/10.3390/molecules28155677/s1, Table S1: Molecular formula and molecular weight of compounds in Figure 2, Figure 3, and Figure 4.
