# Supplementary material for: Gypsogenin Battling for a Front Position in the Pentacyclic Triterpenes Game of Thrones on Anti-Cancer Therapy: A Critical Review—Dedicated to the Memory of Professor Hanaa M. Rady
Source: Molecules. 2023 Jul 27;28(15):5677. doi: 10.3390/molecules28155677 (PMC10420691; doi:10.3390/molecules28155677)
Supplement: Supplementary file 1 [file molecules-28-05677-s001.zip › molecules-2513338-supplementary.pdf]

Gypsogenin Battling for a Front Position in the Pentacyclic Triterpenes *Game of Thrones* on Anti-Cancer Therapy: A Critical Review—Dedicated to the Memory of Professor Hanaa M. Rady

Mohamed O. Radwan [1,2,*], Howaida I. Abd-Alla [2], Azhaar T. Alsaggaf [3], Hatem El-Mezayen [4], Mohammed A. S. Abourehab [5,6], Mohamed E. El-Beeh [7], Hiroshi Tateishi [1], Masami Otsuka [1,8] and Mikako Fujita [1,*]

[1] Medicinal and Biological Chemistry Science Farm Joint Research Laboratory, Faculty of Life Sciences, Kumamoto University, Kumamoto 862-0973, Japan
[2] Chemistry of Natural Compounds Department, National Research Centre, Giza 12622, Egypt
[3] Department of Chemistry, Taibah University, Madinah 42353, Saudi Arabia
[4] Biochemistry Department, Helwan University, Cairo 11795, Egypt
[5] Department of Pharmaceutics and Industrial Pharmacy, Faculty of Pharmacy, Minia University, Minia 61519, Egypt
[6] Department of Pharmaceutics, Faculty of Pharmacy, Umm Al-Qura University, Makkah 21955, Saudi Arabia
[7] Biology Department, Al-Jumum University College, Umm Al-Qura University, Makkah 21955, Saudi Arabia
[8] Department of Drug Discovery, Science Farm Ltd., Kumamoto 862-0976, Japan
* Correspondence: mohamedradwan@kumamoto-u.ac.jp (M.O.R.); mfujita@kumamoto-u.ac.jp (M.F.)

Table S1: Molecular formula and molecular weight of compounds in **Figure 2**, **Figure 3,** and **Figure 4**.

Name	Molecular formula	Molecular weight
**gypsogenin**	$C_{30}H_{46}O_4$	470
**gypsogenic acid**	$C_{30}H_{46}O_5$	486
**1**	$C_{32}H_{48}O_5$	512
**2**	$C_{30}H_{47}NO_4$	485
**3**	$C_{32}H_{49}NO_5$	527
**4**	$C_{37}H_{53}NO_4$	575
**5**	$C_{43}H_{56}N_4O_7$	740
**6**	$C_{37}H_{52}O_4$	560
**7**	$C_{39}H_{54}O_5$	602
**8**	$C_{39}H_{50}F_6O_4$	696
**9**	$C_{38}H_{51}NO_4$	585
**10**	$C_{49}H_{62}O_8$	778
**11**	$C_{50}H_{64}O_9$	808
**12**	$C_{41}H_{62}N_2O_3$	630
**13**	$C_{37}H_{55}NO_3$	561
**14**	$C_{39}H_{59}NO_3$	589
**15**	$C_{37}H_{55}NO_4$	577
**16**	$C_{31}H_{51}NO_3$	485
**17**	$C_{38}H_{57}NO_3$	575
**18**	$C_{34}H_{52}ClNO_4$	573
**19**	$C_{42}H_{67}NO_6$	681
**20**	$C_{35}H_{50}N_2O_4$	562
**21**	$C_{40}H_{65}NO_4$	623
**22**	$C_{37}H_{55}N_3O_4$	605
**23**	$C_{35}H_{50}N_2O_5$	578
**24**	$C_{30}H_{46}O_5$	486
**25**	$C_{30}H_{44}O_5$	484
